# P02.179. Effects of an integrated yoga program on mood, perceived stress, quality of life and immune measures in HIV patients: a pilot study

**DOI:** 10.1186/1472-6882-12-S1-P235

**Published:** 2012-06-12

**Authors:** R Rao, U Deb, N Raghuram, N Hongasandra Rama Rao, A Burke, F Hecht

**Affiliations:** 1 HCG Bangalore Institute of Oncology, Bangalore, Karnataka, India; 2 Swami Vivekananda Yoga Anusandhana Samsthana, Bangalore, India; 3 Osher Center for Integrative Medicine, UCSF, San Francisco, USA

## Purpose

HIV seropositive subjects experience psychological distress that impacts their quality of life and disease progression. In this pilot study we evaluated the effects of a yoga intervention on mood, perceived stress, quality of life and immune responses in HIV+ subjects.

## Methods

Seventy HIV+ subjects not on HAART and with a CD4 count >250 were recruited from a HIV referral center in Bangalore to participate in a two arm randomized waitlist control trial. Subjects were randomized to receive a yoga intervention (N = 36) or serve as wait-list controls (N = 34). While the yoga group received an integrated set of one hour daily yoga therapy sessions (asanas, pranayama and meditation) for 3 months, the waitlist control group received only education and counseling during clinic visits. Both groups were assessed at baseline and after the intervention period using the Hospital Anxiety and Depression Scale, Perceived Stress Scale, the HIV WHO QoL BREF, the Positive and Negative Affect Schedule; CD4, CD8 counts were measured using flow cytometry, and viral load using RT PCR. There were 11 dropouts in yoga and 9 in the control group.

## Results

Data were analyzed using the intention to treat principle. There was a significant decrease in perceived stress (p= 0.001) and psychological distress (p=0.04), and an increase in positive affect (p = 0.003) in the yoga group compared to waitlist controls on ANCOVA with the respective baseline measure as a covariate. There was a decrease in self-report anxiety (p=0.02), depression (p=0.009), negative affect (p=0.02) and fatigue (p=0.001) in the yoga group alone on paired t-test.

## Conclusion

The results suggest benefit with yoga in reducing psychological distress and improving quality of life in HIV seropositive patients. However, larger randomized controlled trials are needed to validate these findings.

